# Characterization of Antibiotic-Loaded Alginate-Osa Starch Microbeads Produced by Ionotropic Pregelation

**DOI:** 10.1155/2013/472626

**Published:** 2013-06-03

**Authors:** Gizele Cardoso Fontes, Verônica Maria Araújo Calado, Alexandre Malta Rossi, Maria Helena Miguez da Rocha-Leão

**Affiliations:** ^1^Escola de Química, Universidade Federal do Rio de Janeiro, 21949-900 Rio de Janeiro, RJ, Brazil; ^2^Centro Brasileiro de Pesquisas Físicas, Rua Dr. Xavier Sigaud 150, 22290-180 Urca, RJ, Brazil

## Abstract

The aim of this study was to characterize the penicillin-loaded microbeads composed of alginate and octenyl succinic anhydride (OSA) starch prepared by ionotropic pregelation with calcium chloride and to evaluate their *in vitro* drug delivery profile. The beads were characterized by size, scanning electron microscopy (SEM), zeta potential, swelling behavior, and degree of erosion. Also, the possible interaction between penicillin and biopolymers was investigated by differential scanning calorimetry (DSC), powder X-ray diffraction (XRD), and Fourier transform infrared (FTIR) analysis. The SEM micrograph results indicated a homogeneous drug distribution in the matrix. Also, based on thermal analyses (TGA/DSC), interactions were detected between microbead components. Although FTIR spectra of penicillin-loaded microbeads did not reveal the formation of new chemical entities, they confirmed the chemical drug stability. XRD patterns showed that the incorporated crystalline structure of penicillin did not significantly alter the primarily amorphous polymeric network. In addition, the results confirmed a prolonged penicillin delivery system profile. These results imply that alginate and OSA starch beads can be used as a suitable controlled-release carrier for penicillin.

## 1. Introduction

Benzathine penicillin G (PenG) and its semisynthetic derivatives comprise one of the main groups of antimicrobials used in the treatment of major infectious diseases [1]. Since its discovery in the 1920s, penicillin has been administered through injection. The discomfort and inconvenience associated with this type of administration have led patients with rheumatic fever to neglect and even stop the therapy [2]. For this reason, alternative procedures of PenG administration using polymeric matrices for controlled release systems, such as poly-D,L-lactic acid-co-glycolic acid polymer (PLGA) [3], polyethylene glycol polyamidoamine (PEG-PAMAM) star polymer [4], polybutyl adipato (PBA) [5], and polyacrylate [6], have been proposed. 

The use of natural polymers in the design of drug delivery formulations has received much attention because of their excellent biocompatibility and biodegradability. Among them, alginate and starch are very promising and have been exploited in the pharmaceutical industry for controlled drug delivery. 

Alginates are composed of (1-4)-linked *β*-D-mannuronic acid (M units) and *α*-L-guluronic acid (G units) monomers, which vary in amount and sequential distribution along the polymer chain depending on the source of alginate [7]. Divalent cations, such as Ca^2+^, cooperatively bind between the G-blocks of adjacent alginate chains, producing the so-called “egg-box” structure and creating ionic interchain bridges, which cause gelling of aqueous alginate solutions [8]. 

Another potential biopolymer for drug delivery is starch. Starch is regenerated from carbon dioxide and water by photosynthesis in plants. Owing to its complete biodegradability, low cost, and renewability, starch is considered a promising candidate for developing sustainable materials [9]. Octenyl succinic anhydride (OSA) starch is a modified starch developed by the National Starch and Chemical Corporation in the United States. This modification consists of the addition of a lipophilic component (octenyl-succinate), which increases the emulsion stability in formulations [10]. Furthermore, it is a polymer applied widely in controlled release systems because it can lead to the formation of less porous materials. 

Mixtures of alginate and OSA starch have been studied as coating materials for ascorbic acid edible film [10]. This application was the first example in which a calcium alginate-OSA starch mixture was made to obtain microbeads containing antibiotic with potential for biomedical applications. This combination is noteworthy because the calcium alginate forms a reticulate polymer and the OSA starch may contribute to decreasing the pore size of this polymeric matrix to allow the retention of many amphipathic components within the material, such as PenG, because of its both hydrophilic and hydrophobic nature. 

Fontes et al. [11] developed and optimized PenG microencapsulation from alginate and OSA starch biopolymers, obtaining a high retention percentage of antibiotic in the microbeads. Thus, in the present work, we have performed an in-depth characterization of microbeads developed by Fontes et al. [11] for future application in subdermal implants using several different techniques. Understanding both the correlation between physical and chemical properties of the structural characteristics and the drug release kinetics are of crucial importance for the development of new products.

We have analyzed the thermal and structural properties and drug release kinetics of the microspheres and biopolymers in an attempt to gain a better understanding of the structure-function relationship. The thermal properties were characterized using thermogravimetric analysis (TGA) and differential scanning calorimetry (DSC). The structure of the beads was studied using scanning electron microscopy and X-ray diffraction (XRD). The chemical propriety was analyzed by FTIR studies. The swelling and erosion behaviors were also evaluated because of their effects on the diffusion and release of drugs when microspheres are applied in drug delivery systems. Comprehensive knowledge of the material properties of a product in production and its application conditions is critical for a product success.

## 2. Materials and Methods

### 2.1. Materials

Sodium alginate was obtained from Keltone LV. The ratio of mannuronic acid to guluronic acid residues (M/G) ranged from 0.4 to 1.9. Octenyl succinic anhydride (OSA) starch was obtained from National Starch, and CaCl_2_·H_2_O was obtained from Vetec. Penicillin G sodium salt was purchased from Sigma-Aldrich (Fluka and Science Lab, USA).

### 2.2. Sample Preparation

The beads containing PenG were prepared in triplicate by the extrusion method (dripping method) as described by Fontes et al. [11]. Sodium alginate (4,5%, w/v) with OSA starch (2%, w/v) and PenG (10%, w/v) was dissolved in distilled water. The solution was stirred thoroughly for 30 minutes to ensure complete mixing of the drug. The mixture was dropped into calcium chloride solution (2%, w/v). After gelation, the beads remained in the CaCl_2_ solution for 10 min. The beads were then removed, washed with Milli-Q water, and dried in the oven at 35°C for 12 h. 

### 2.3. Retention Percentage of PenG Entrapment in Beads

The retention percentage of PenG was determined by HPLC analysis (*γ* = 220 nm, Waters). The alginate-OSA starch beads loaded with PenG were completely dissolved in phosphate buffer pH 7.4 and measured by HPLC. Preliminary UV studies showed that the presence of dissolved polymers did not interfere with the absorbance of the drug at 220 nm. HPLC analysis of PenG was performed using a Waters 1525 HPLC system with an analytical 4.6 × 150 mm (5 *μ*m) RPC18 YMC-Pack ODS-AQ column, a flow rate of 1 mL/min, and a detector wavelength of 220 nm (Waters 2414). Samples (15 *μ*L) were injected and eluted using A (0.025 M KH_2_PO_4_ in water (pH = 3)) and B (acetonitrile) as the mobile phase with an isocratic method (67% of A). PenG showed a retention time of 4.5 ± 0.17 min, and its detection limit was 0.1 *μ*g/mL. The retention percentage (%) of entrapment PenG was calculated using the following equation:

(1) Retention (%)of  entrapment   =mass  of  penicillin  present  in  beadsmass  of  penicillin  present  in  the  formulation×100.

### 2.4. *In Vitro* PenG Release Studies

The *in vitro* PenG release profiles of the beads were followed in 20 mL of Milli-Q water for 800 h. without mechanical stirring at 37°C. At predetermined time intervals, 0.7 mL of the samples was withdrawn and replaced with fresh medium. The PenG content was determined by HPLC at 220 nm. The unloaded OSA starch alginate beads (without PenG) were taken as reference. Each experiment was done in triplicate.

#### 2.4.1. Analysis of Release Data

To analyze the *in vitro* release data, various kinetic models were used. The zero-order rate equation (2) describes the systems where the drug release rate is independent of its concentration [12]. The first-order equation (3) describes the release from the system where the release rate is concentration dependent [13]. Higuchi [14] described the release of drugs from an insoluble matrix as the square root of the time-dependent process based on Fickian diffusion (4). The Baker-Lonsdale model (5) describes the drug release from spherical matrices [15]. Korsmeyer et al. [16] derived a simple relationship that described drug release from a polymeric system equation (6). The adjusted coefficient of determination (*R*_adjusted_^2^) value was used as a criterion to choose the best model to describe drug release:

(2) $$\begin{array}{c} \frac{Q_{t}}{Q_{\infty }}=K_{0}t+Q_{0}, \end{array}$$

(3) $$\begin{array}{c} \log Q_{t}=\log Q_{0}-\frac{K_{1}t}{2,303}, \end{array}$$

(4) $$\begin{array}{c} \frac{Q_{t}}{Q_{\infty }}=K_{h}\sqrt{t}, \end{array}$$

(5) (32)[1−(1−(QtQ∞))2/3]−(QtQ∞)=Kbt,

(6) $$\begin{array}{c} \frac{Q_{t}}{Q_{\infty }}=K_{k}t^{n}+Q_{0}, \end{array}$$

where *Q*_*t*_/*Q*_*∞*_ is the amount of drug released in time *t*, *Q*_0_ is the initial amount of the drug, *K*_0_ is the zero-order rate constant, *K*_1_ is the first order constant, *K*_*h*_ is the rate constant for Higuchi, *K*_*b*_ is the rate constant for Baker-Lonsdale, and *K*_*k*_ is the rate constant for Korsmeyer Peppas.

### 2.5. Characterization

#### 2.5.1. Swelling Behavior

Swelling studies were conducted using both wet and dry beads. The term wet refers to the state of the beads immediately after the preparation, and the term dry refers to beads that were left to dry for 24 h. at 37°C in air. Swelling studies of alginate-OSA starch-PenG beads were carried out in Milli-Q water media. The samples were placed in water, and the weight of the swollen samples was measured over time after the excess surface water was removed by gently tapping the surface with a dry piece of filter paper. The degree of swelling (*S*_*W*_) for the beads sample at time *t* was calculated using (7):

(7) $$\begin{array}{c} S_{W}\left(\%\right)=\left(\frac{W_{S}-W_{i}}{W_{i}}\right)\times 100, \end{array}$$

where *W*_*S*_ is the weight of the beads in the swollen state and *W*_*i*_ is the initial weight of the dry beads.

#### 2.5.2. Erosion Determination

The degree of erosion was determined after the immersion of dry beads in 20 mL of Milli-Q water. After a selected time interval, the beads were withdrawn and dried in an oven at 110–120°C for a 24 h time period, allowed to cool in a desiccator, and finally weighed until constant weight was achieved (final dry weight). Three different samples were measured for each time point, and fresh samples were used for each individual time point. The percentage erosion (*E*) was estimated as follows:

(8) $$\begin{array}{c} E\left(\%\right)=\frac{W_{i}-W_{f}}{W_{i}}\times 100, \end{array}$$

where *W*_*i*_ is the initial starting dry weight and *W*_*f*_ is the final weight of the same dried and partially eroded sample. All experiments were done in triplicate.

#### 2.5.3. Scanning Electronic Microscopy and Zeta Potential

The microstructures of the beads were studied by scanning electron microscopy (SEM). Randomly selected dry beads were deposited on double-coated carbon conductive tape previously adhered to SEM aluminum stubs. The bead samples were then sputter-coated with a thin gold layer using a coating unit (Balzers Union model FL 9496) and analyzed in a JEOL JSM 5310 operated at 15 or 20 kV. For the surface morphology observation, beads with and without PenG addition were utilized.

The surface charge of beads was evaluated as a function of pH by zeta potential (Dispersion Technology DT 1200).

#### 2.5.4. X-Ray Diffraction (XRD)

X-diffraction patterns of the individual biomaterials, physical mixtures, and beads were obtained with an X-ray diffractometer (PANalytical, model X'Pert PRO) using a K*α*Cu radiation wavelength of 1.54184 Å [17].

#### 2.5.5. FT-Infrared Spectroscopy

Individual beads/samples were crushed in a mortar with a pestle. The crushed material was mixed with potassium bromide in a 1 : 100 proportion and dried at 40°C. The mixture was compressed to a 12 mm semitransparent disk by applying a pressure of 10 tons for 2 minutes. The FTIR spectra over the wavelength range from 4100 to 500 cm^−1^ were recorded using an FTIR spectrometer (Prestige 21-Shimadzu).

#### 2.5.6. Thermal Analysis

*Differential Scanning Calorimetry (DSC)*. The DSC of PenG, sodium alginate, OSA starch, as well as unloaded alginate/OSA starch beads, and PenG/sodium alginate/OSA starch beads were evaluated. The test was carried out by using a thermal analysis system (Perkin-Elmer, Diamond) calibrated with indium as the standard and operated in the temperature range of 30–400°C. The bead sample (5 mg) was heated at a rate of 10°C min^−1^ in an aluminum pan under a nitrogen atmosphere using an empty pan as the reference. The onsets of melting point and enthalpy of fusion were automatically computed.

*Thermogravimetric Analysis (TGA)*. Degradation temperatures were performed in a TGA (Perkin-Elmer, Pyris 1) using a sample mass of *ca.* 7 mg and an platinum sample holder at a heating rate of 10°C min^−1^ under a dynamic nitrogen atmosphere flowing at 20 mL min^−1^. 

## 3. Results and Discussion

### 3.1. Morphological Characterization, Size, and Surface Charge

In this study, microbead drug delivery systems were prepared using alginate and OSA starch as wall materials for PenG microencapsulation. Because both PenG and alginate are electronically negative and Ca^2+^ is electronically positive, polyelectrolyte complexes can be formed between PenG/alginate and Ca^2+^ via electrostatic interactions during the microparticle preparation. Furthermore, the interactions between PenG and OSA starch are favorable, as PenG has a hydrophobic portion that can interact with the octenyl-succinate lipophilic component of OSA starch. The dripping technique produced spherical droplets that, after falling into the CaCl_2_ solution, resulted in spherical thermostable gel particles because of the ionic interactions between guluronate blocks formed from alginate and Ca^2+^ ions.

The results showed a high retention percentage of PenG (95.4%) when alginate and OSA starch were used as the wall materials [11].

**Figure 1:**
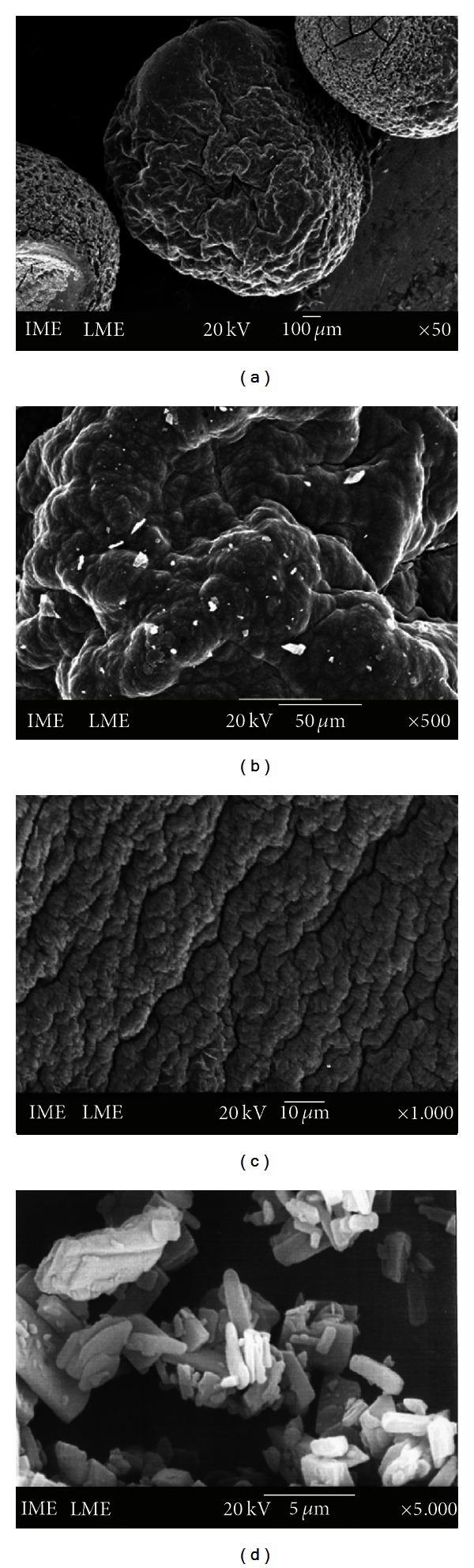
SEM photomicrographs of OSA starch/alginate containing PenG (a), a section near the surface of the bead (b), beads without PenG (c), and pure PenG (d).

PenG-loaded alginate/OSA starch beads showed a spherical geometry (1 ± 2 mm diameter) and a compact structure as evidenced by the SEM analysis reported in Figure 1.

The surface of the beads exhibited a homogeneous microstructure with several wrinkles. The arrangement of the particles formed a surface porosity with pores of a few micrometers in diameter.  Figure 1(b) shows a section near the surface of the bead. Some crystals of PenG were observed at the surface of the beads.  Figure 1(d) shows pure PenG micrographs. The image reveals the existence of elongated and radially oriented crystals.

Figure 1(c) represents the surface micrographs for beads without PenG, in which a cohesive and compact surface arrangement was observed with irregularities that include peaks and troughs. The irregularities can be attributed to the drying process and the cohesions may be because of the ionic and electrostatic interactions between the components of the beads. The morphology observed is typical of drug-loaded alginate beads, similar to that observed by Rajendran and Basu [17] and Liu et al. [18]. 

**Figure 2:**
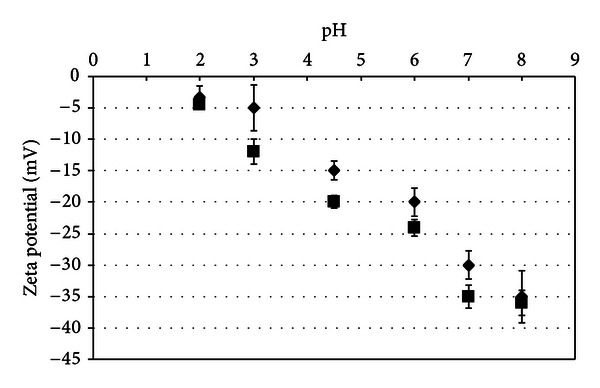
Zeta potential of unloaded alginate/OSA starch beads (♦) and PenG alginate/OSA starch beads (■) as a function of pH.

The surface charge of the beads was evaluated as a function of pH (Figure 2). The results from the zeta potential evaluation showed that the surface potential of the microbeads was negatively charged. A greater negative value of the zeta potential was obtained upon an increase in pH, which changed regularly from −3 to −35 mV. In the low pH region (pH 2), most of the carboxylic acid groups in the alginate and OSA starch were in the form of –COOH because the pKa of alginate is in the range of 3.4 to 4.4 [19] and OSA starch is approximately 4.76 [20]. 

When the pH of the medium was increased, the carboxylic acid groups became ionized, resulting in the increase of negative charge. The PenG alginate/OSA starch beads presented higher negative values of zeta potential than the unloaded beads. This observation may be attributed to the negativelycharged PenG on the alginate/OSA starch microbeads, which caused a decrease in zeta potential. This result shows that beads can make ionic interactions with other biomacromolecules that have a positive charge, such as chitosan and polylysine.

### 3.2. Swelling Behavior and Erosion Determination

When hydrophilic polymers come into contact with a liquid hydrate, a gel layer is formed. The formation of the gel layer is essential for sustaining and controlling drug release from polymer solid dosage forms. The thickness of this hydrated layer determines the diffusion of the drug molecules through the polymer mass into the liquid medium, but diffusion is not the only mechanism controlling drug release. The rate and extent of drug release also depend on the swelling and erosion of the hydrated polymer preparation [21].

**Figure 3:**
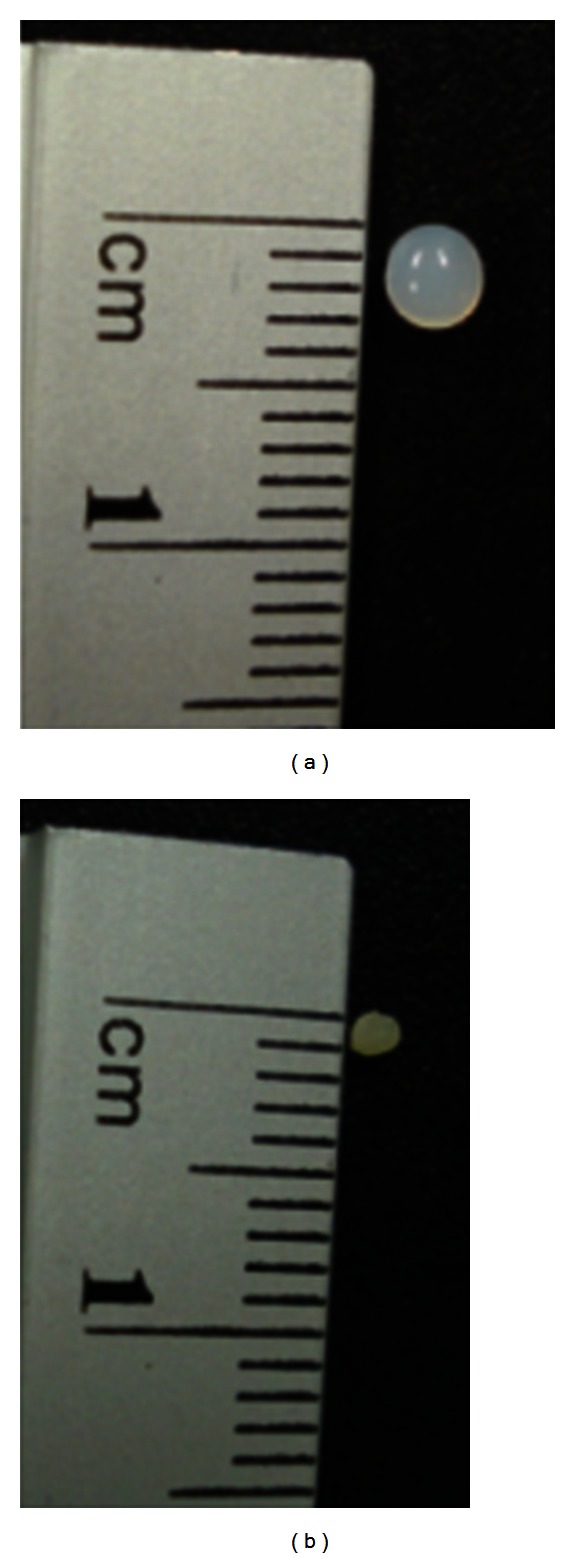
Photograph of wet beads taken after preparation (a) and dry beads (b).

Figure 3(a) shows the beads immediately after preparation, and Figure 3(b) shows the dry bead. In comparison with the size of the wet beads, which was measured to be 3.0 ± 0.2 mm, dried beads were shrunken and their diameter was found to be 1.2 ± 0.09 mm. The maximum degree of swelling calculated is 637.75%. The swelling behavior can be explained by the fact that wet beads tend to absorb water (free or bulk water) in order to fill the empty regions of the polymer network within the beads until they reach the equilibrium state [22].

**Figure 4:**
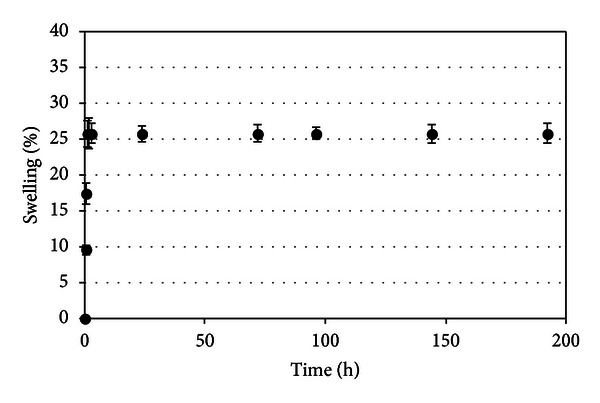
Swelling profiles of dry beads in water. Values are expressed as mean ± standard deviation (S.D.) of three experiments.

Figure 4 shows the swelling behavior of dry beads in water. The results obtained using dry beads varied substantially when compared to the wet beads in terms of maximum swelling degree, which was lower by approximately one order of magnitude. The swelling of the beads reached 25% in the first 24 hours and then remained constant until 960 hours. The swelling of the dry beads is mainly attributed to the hydration of the hydrophilic groups of alginate [23]. In this case, free water penetrates inside the beads to fill the inert pores among the polymer chains, which contributes to the degree of swelling. In the literature, different swelling degrees (from 80% to 220%) were observed when alginate was used as the wall material. The lower swelling degree observed in the present work was mainly due to the intrinsic rigidity of the polysaccharide as well as the extent of cross-linking between the carboxyl groups of alginate, Ca^2+^, and OSA starch. When dried, the alginate network shrinks and collapses, and egg-box junctions move close enough to form side-by-side aggregations under the mediation of free calcium ions. This process is equal to the increasing cross-linking density and makes the dried alginate gel structure extremely dense, which is the reason that the alginate gel beads barely swelled in pure water, as shown in Figure 4.

According to Davidovich-Pinhas and Bianco-Peled [23], the degree of swelling diminishes considerably as calcium concentration increases. An increase of calcium concentration probably increases the number of alginate strands creating a thicker “egg-box” model, which results in a stronger network and hinders water absorption [24]. Therefore, the cross-linking conditions used in this work (previously optimized by Fontes et al. [11]) were sufficient to promote considerable cohesion between the polymers, avoiding a higher water uptake. 

**Figure 5:**
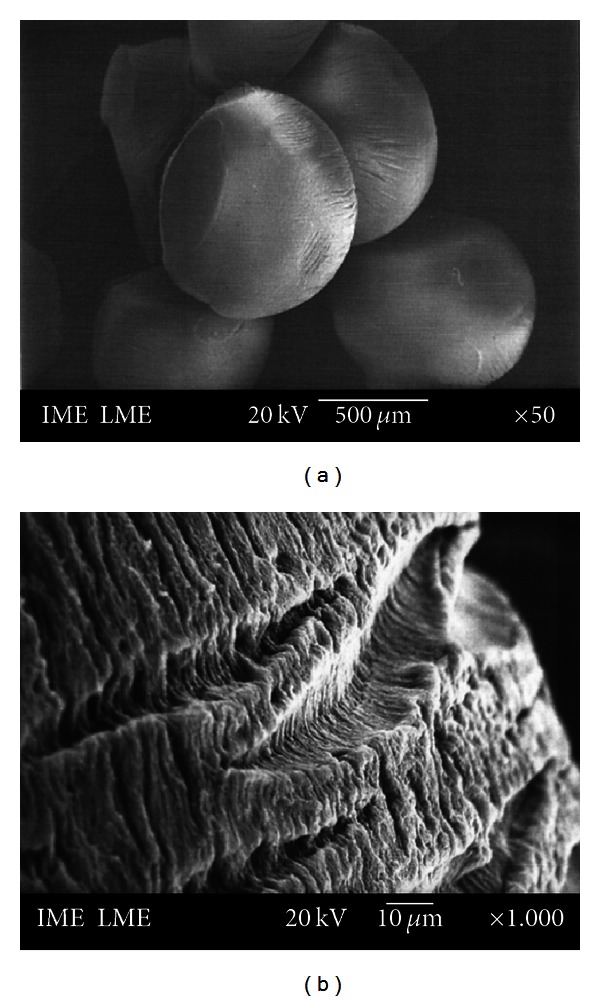
SEM photomicrographs of OSA starch/alginate beads taken after 30 days of water immersion.

The beads did not show any visual sign of disintegration in the media over a period of 30 days.  Figure 5 presents the scanning electron micrographs of the beads after 30 days of immersion in water. There was a change in the morphology of the beads, as seen in Figure 5. The surface of the beads was smoother as compared to the micrographs shown in Figure 1. Irregular pores and cracks were observed on the beads, probably due to erosion of the polymer matrix. 

Another parameter evaluated was the degree of bead erosion. A decrease in the weight of the beads occurred after 24 hours of water immersion when the maximum degree of hydration was reached. The hydration probably caused the removal and solubilization of the polymer chains with a slight loss in the weight of the polymers. The maximum erosion degree of the beads was 5% over 30 days of experiments.

### 3.3. *In Vitro* Drug Release Kinetics

**Figure 6:**
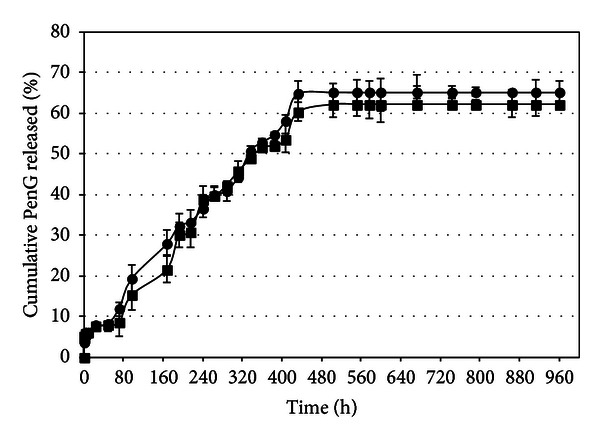
PenG release from alginate and OSA starch beads in Milli-Q water (*⚫*) and (■) 0.35 M calcium chloride without mechanical stirring at 37°C.

The release behavior of PenG-loaded beads is presented in Figure 6. The purpose of these drug delivery systems is to implant the microbeads subcutaneously. For that reason, water was chosen as the release medium instead of simulated gastric fluid or even a phosphate buffer, which has an affinity for calcium and could influence the PenG release by alginate erosion. The release of PenG from alginate beads in Milli-Q water was monitored periodically until its concentration in the solution reached a constant value. It was verified (Figure 6) that the PenG release occurs in at least two steps: first, a fast release of about 5% in the first hour of the assay, which corresponds to PenG being physically entrapped in the bead's external layer, and second, the PenG being gradually released, reaching 65% in 432 hours. This second stage can be attributed to the diffusion of the antibiotic from the bead's interior to the outside. After 432 h of assay in this experiment, the PenG concentration was constant until the end (~960 h). This multistage release pattern is due to the complexity of the bead's microstructure, as presented earlier. 

The kinetics of PenG release in alginate and OSA starch beads was also evaluated in a 0.35 M calcium chloride solution in order to investigate whether increasing ionic strength by the presence of calcium ions would influence the release of PenG. Figure 6 shows that there is no difference in the release profile when a different medium released is used. 

In order to understand the kinetics and mechanism of drug release, the *in vitro* release data were studied using various kinetic models to predict the drug release kinetic mechanism.  Table 1 shows the release parameters of PenG sustained release and adjusted coefficient of determination (*R* ^2^) values in various kinetic models tested. The release kinetics of PenG was found to be better described by the zero-order equation, which provided good linearity and the best fit line (*R*_adjusted_^2^ = 0.99).

According to Kiortsis et al. [25], for the controlled release under investigation and a hydrophilic polymer, the release should follow three steps. The first step is the penetration of the dissolution medium in the matrix (hydration). The second step is the swelling with concomitant or subsequent dissolution or erosion of the matrix. The third step is the transport of the dissolved drug, either through the hydrated matrix or from the parts of the eroded tablet, to the surrounding dissolution medium.

By incorporating the first 60% of release, data mechanism of release can be indicated according to Korsmeyer, where *n* is the release exponent, indicative of mechanism of drug release. For the case of sphere shape, 0.43 ≤ *n* corresponds to Fickian diffusion mechanism, 0.43 < *n* < 0.85 to anomalous (non-Fickian) transport, *n* = 0.85 to Case II (relaxation) transport, and *n* > 0.85 to super Case II transport (Siepmann and Peppas, 2001). The value of the release exponent in PenG sustained release obtained was 0.82. This indicates that PenG release from alginate-OSA starch beads followed anomalous transport. In the anomalous processes of drug release, Fickian diffusion through the hydrated layers of the matrix and polymer chain relaxation/erosion are both involved [26]. 

For predictive completion of release, the drug release data of PenG obtained from dissolution is plotted as concentration (mg·L^−1^) versus time (*h*). Linear regression analysis of the data yields the equation of best line as *c* = 6.45*t* + 190.67 and *R* ^2^ = 0.99. 

According to (2), the slope of line corresponds to the zero-order rate constant. Therefore, the rate of dissolution is *k*_0_ = 6.45 mg·L^−1^ h^−1^. The rate of release in terms of amount of PenG dissolved or released per unit time can be obtained as follows:

(9) $$\begin{array}{c} k_{0}\times V, \end{array}$$

where *V* is volume of dissolution medium (*L*).

The rate of release calculated from (9) is 0.13 mg·h^−1^. Assuming that this rate of release remains constant throughout the release process, the duration of release is calculated as 769.23 h (32 days) using 100 mg. Hence, the formulation allows a gradual release of the antibiotic from the beads. PenG diffuses through an outer gel layer, which erodes and allows the aqueous medium to penetrate further into the core.

### 3.4. The X-Ray Diffraction

**Figure 7:**
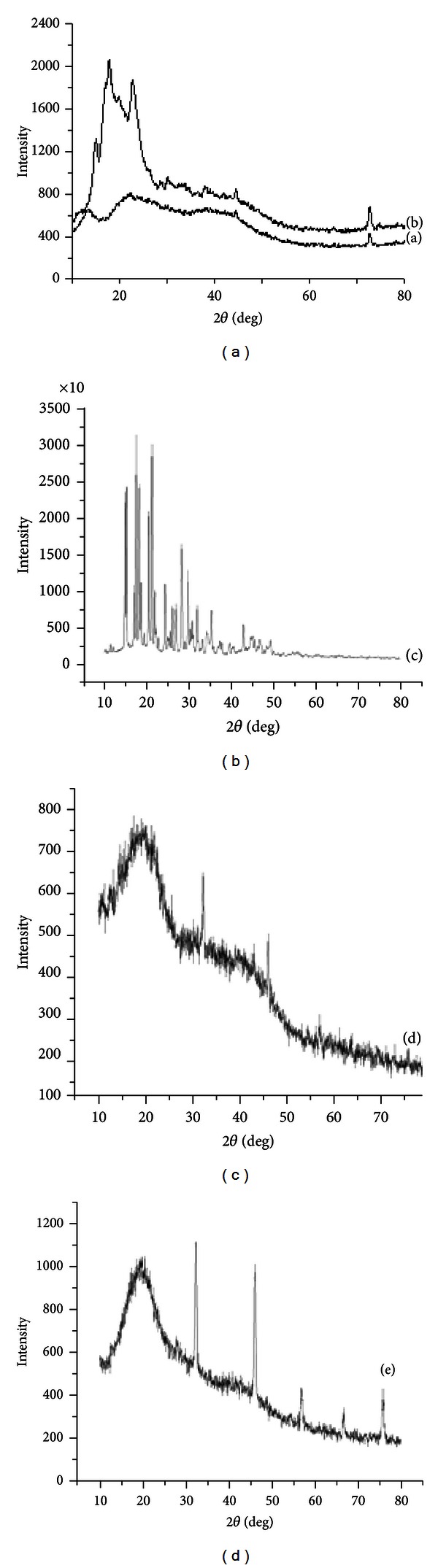
X-ray powder diffraction patterns of sodium alginate (a), OSA starch (b), PenG (c), physical mixture of OSA starch with sodium alginate (d), and PenG-loaded alginate/OSA starch beads (e).

The X-ray diffraction patterns of the individual polymers, physical mixtures of the polymers, PenG alone, and the PenG-loaded beads are displayed in Figure 7. The diffractogram of alginate consisted of three crystalline peaks at 2*θ* = 13.7°,  23.0°, and 40°. However, according to Wang et al. [27] and Yang et al. [28], the alginate X-ray diffraction consisted of only two crystalline peaks at 2*θ* = 13.7° and 23.0°. This difference may be because of the amounts of guluronic and manuronic acid present in the different alginate samples. OSA starch also showed three crystalline peaks at 2*θ* = 16°, 18°, and 22°. It presented a typical semicrystalline structure because of its close molecular packing and regular crystallization. Also, Tukomane et al. [29] state that the starch crystalline region is an ordered arrangement of double helical amylopectin structures. Amylose is associated with the amorphous regions and is responsible for water uptake, which occurs more readily at temperatures below that of gelatinization temperature. Pure PenG showed a typical diffractogram of the crystalline substance, with intensive peaks between 17° and 40°. The degree of PenG crystallinity decreased as observed in the X-ray diffraction patterns of the PenG-loaded beads. This decrease can be explained by the possibility of the drug distribution over the polymeric matrix. It is believed that the drug presents some molecular mobility capacity among the polymeric chains, characterizing the observed semiamorphous state of mixture. In addition, the crystallinity of OSA starch was not observed in the beads due to the strong interaction between alginate and OSA starch. This interaction destroyed the close packing of the polymer molecules required for the formation of regular crystallites. Similar results were observed by Wang et al. [27] when alginate and starch were mixed for fiber production. 

The X-ray diffraction patterns of the physical mixtures of polymers and beads showed new peaks of crystallinity at 2*θ* = 32° and 46°. The regularity of the crystal structure may be because of the egg-box regions along the direction of the alginate chain and its aggregating direction.

### 3.5. Thermal Analysis

Thermal analysis is the most common approach to study physicochemical interactions of a two- or more component system [30].  Table 2 presents the endothermic and exothermic peaks and enthalpies associated with each peak for alginate, OSA starch, unloaded alginate/OSA starch beads, PenG, and PenG/alginate/OSA starch beads by DSC. 

Alginate and OSA starch presented one endothermic peak, which is probably due to the melting point. The exothermic peaks of alginate at 237°C resulted from the decomposition of biopolymer because of depolymerization reactions, which are likely owing to the partial decarboxylation of the protonated carboxylic groups and oxidation reactions of the polyelectrolytes [31]. 

The PenG thermograms presented two exothermic peaks, which are possibly attributed to oxidation. 

When alginate was mixed with OSA starch for the production of beads by ionotropic gelation, the thermogram also showed one endothermic peak. The endothermic peak shifted to a higher temperature (196.08°C). This shift corresponded to the interaction of alginate with OSA starch and calcium ions, which showed higher stability of the complexes. The degradation exothermic peak of sodium alginate was absent in the beads; thus, no decomposition event was observed up to 350°C. The thermograms of the PenG-loaded alginate-OSA starch beads did not present any peak during analyses. These results indicate good interaction between both components.

The glass transition temperatures (*T*_*g*_) were also determined. This parameter can be used as a measurement for the mobility of the macromolecules and the evaluation of the solid-to-liquid transition [32]. The *T*_*g*_ of alginate and OSA starch were 52.10, 67.06°C, respectively (Table 2). Segura-Campos et al. [32] reported that OSA starch *T*_*g*_ was 64.6°C. Similar alginate *T*_*g*_ values were found in the literature [33]. The *T*_*g*_ value of unloaded beads is similar to altinate *T*_*g*_ value due to higher amount of alginate in the beads. PenG did not present *T*_*g*_, which is likely explained by its structure crystalline. The antibiotic alginate/OSA starch beads show a higher *T*_*g*_ value (80.51°C), which indicates a predominant energetic interaction between the biopolymers and the antibiotic. This effect causes an increase of the glass transition due to the denser packing in the mixture because of the decreasing mobility and the free volume caused by the local ordering effect of heterocontact formation [34]. 

Brekner et al. [35] have suggested that the glass transition temperature of compatible polymer mixtures depends on the free volume distribution and the related conformational mobility, which is controlled by the probability of heteromolecular contact in the mixture due to specific interactions of the components.

**Figure 8:**
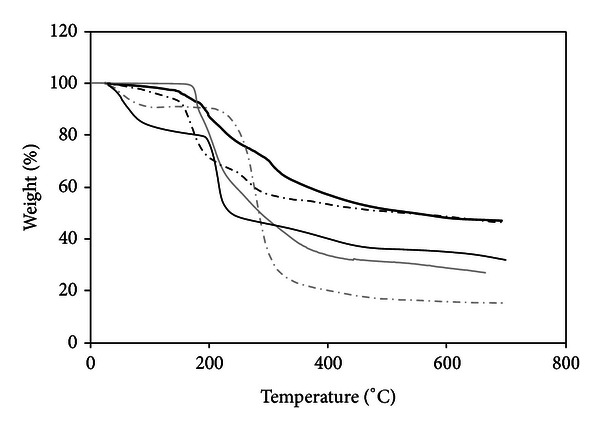
Thermogravimetric curves of alginate (—), OSA starch (dashed grey line), PenG (grey line), alginate/OSA starch microspheres (dashed black line), and PenG/alginate/OSA starch microbeads (black line).

Another analysis carried out was thermogravimetry (TGA). TGA has been proved to be a suitable method to investigate the thermal stability of polymeric systems. The knowledge of the threshold decomposition temperature and mode of decomposition upon heating is recommended to determine the highest processing temperature that can be used [36]. The TGA results of the studied materials are shown in Figure 8. 

The alginate TG curve presents an initial dehydration process followed by decomposition in two overlapping steps under nitrogen, which is in agreement with DSC data. Similar behavior was observed by Soares et al. [33]; the decomposition product around 400°C was characterized as a carbonaceous material.

The initial weight loss of OSA starch that began just above room temperature corresponds to water desorption. This process was not observed in the mixtures, an indication that a small quantity of water is adsorbed in their structures. OSA starch shows a single degradation step at 250°C.

The unloaded alginate/OSA starch beads showed a two-step degradation inherent to both biopolymers. PenG TGA curves show that weight loss occurred only after 200°C in a single-step degradation.

The OSA starch, alginate, and PenG exhibited 84.92, 68.2, and 73.49% of weight loss at 500°C, while the unloaded alginate-OSA starch beads and PenG/alginate/OSA starch beads presented 53.55% of weight loss at 500°C. The lower weight loss of the beads could be the result of strong interactions between the components of the beads. The thermal stability at 100–120°C (the temperature used in the microbead preparation) shows that both samples were stable during the preparation of the beads, indicating the viability of the implant preparation method developed.

### 3.6. The Fourier Transform Infrared Spectroscopy (FTIR) Characterization

**Figure 9:**
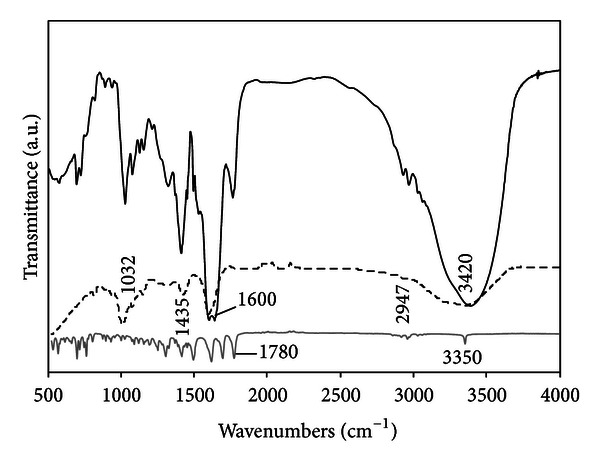
The FTIR spectroscopy of unloaded alginate/OSA starch beads (dashed line), PenG (grey line), and PenG/alginate/OSA starch microspheres (black line).

The possibility of chemical interactions was evaluated by FTIR.  Figure 9 shows the infrared spectra of PenG, unloaded alginate/OSA starch beads, and PenG-loaded alginate/OSA starch beads.

The FTIR spectra of unloaded alginate/OSA starch beads provide evidence of both alginate and OSA starch structure information. For the alginate, characteristic functional groups (COO^−^ stretching) were present, with a broad asymmetrical band at 1610 cm^−1^ and a narrower symmetrical band at 1418 cm^−1^. An even broader absorption was observed near 1030 cm^−1^, which can be attributed to COH stretching. OSA starch has similar profiles. In the fingerprint region, there are several discernible absorbances at 1155, 1080, 1021, and 930 cm^−1^, which were attributed to CO bond stretching [37]. The band at 2928 cm^−1^ is characteristic of the CH stretching vibration. An extremely broad band resulting from vibration of the hydroxyl groups (OH) appeared at 3390 cm^−1^. These results were confirmed by FTIR spectra obtained from both pure alginate and OSA starch (data not shown) and by the previously reported literature [38].

The FTIR spectra of PenG alone showed major peaks in the wave number ranging from 800 to 1500 cm^−1^, indicating the presence of carboxyl and carboxylate group stretching. The band at 3350 cm^−1^ was assigned to the NH stretching. The mode oxazolone showed one characteristic band at 1790 cm^−1^ for the carbonyl group. The thiazolidine structure in PenG displayed the carbonyl band at 1622 cm^−1^. The bands at 1502 and 1701 cm^−1^ are characteristic of primary amide and secondary amide structures, respectively [39].

In FTIR spectra of PenG-loaded alginate/OSA starch beads, no extra bindings or chemical shifts were observed, indicating that there is no strong chemical interaction between polymer and drug into the polymer/drug network. Although DSC data show that there are physical interactions, as mentioned earlier, between the polymer and drug, the FTIR spectra did not reveal the formation of new chemical entities. These results confirm the drug's chemical stability, the permanence of its biological activity, and the possibility of a sustained drug delivery system profile. 

## 4. Conclusions 

In the present work, antibiotic-loaded alginate/OSA starch microbeads were successfully characterized using several different techniques. The morphological characterization showed that the homogeneous microbeads had negative surface charge. We have shown, by XRD, PenG distribution onto the polymeric matrix. These findings were confirmed by DSC. Shifts of the endothermic and exothermic peaks observed between individual biopolymers and final microbead carriers were interpreted as interactions with different thermal properties. Also, FTIR analysis showed the absence of detectable chemical interactions between the drug and polymer and the presence of PenG in structural biological activity. The *in vitro* release studies proved the capacity of the microbeads to release the drug in a prolonged profile, and the release kinetics of PenG was found to be better described by the zero-order equation. In summary, the delivery system developed and fully characterized in the present work can be applied as a subdermal implant for the treatment of rheumatic fever and other antimicrobial treatments in the future.

## Figures and Tables

**Table 1:** Release parameters (*K*) of PenG sustained release and adjusted coefficient of determination (*R* ^2^) values in various kinetic models tested.

Zero order		First order		Higuchi				Korsmeyer Peppas		
Cumulative release % versus time (day)		log % drug remaining versus time (day)		Cumulative % drug release versus square root of time (day)		Baker-Lonsdale		log % cumulative versus log time (day)		
*R* ^2^	*K*	*R* ^2^	*K*	*R* ^2^	*K*	*R* ^2^	*K*	*R* ^2^	*n* value	*K*
0.99	3.55 ± 0.07	0.83	0.12 ± 0.012	0.97	12.60 ± 0.39	0.92	0	0.96	0.82	5.63 ± 0.71

**Table 2:** Peak temperatures and enthalpy changes in the DSC thermograms collected from alginate, OSA starch, unloaded alginate/OSA starch beads, PenG, and PenG-alginate-OSA starch beads.

Sample	Temperature (°C)			Δ*H* (J/g)	*T*_*g*_
	Onset	Peak	Endset		
Alginate	161.40	163.26	170.33	332.98	52.10
	209.59	237.36	263.35	−359.40	
OSA starch	182.65	184.31	189.55	125.03	67.06
Unloaded alginate/OSA starch beads	145.57	196.08	220.65	661.07	51.21
Penicillin	189.31	210.52	224.82	−70.69	—
	224.82	226.63	250.00	226.63	
Penicillin/alginate/OSA starch beads	—	—	—	—	80.51
